# Supplementary material for: Minimal Input, Maximum Insight: Inferring Material Parameters From Solar Cell JV Curves Alone
Source: Small Methods. 2026 Aug 11;10(18):e70921. doi: 10.1002/smtd.70921 (PMC13599703; doi:10.1002/smtd.70921)
Supplement: Supplementary file 1 [file SMTD-10-e70921-s001.pdf]

Supplemental Information

S1. IMPLEMENTED NETWORKS

A. Properties Varied

Property	Parameter	Minimum	Maximum	Lin/Log	Units
Free electron mobility	Electron mobility y	1×10^{-10}	1×10^{-5}	Log	$m^2V^{-2}s^{-1}$
Free hole mobility	Hole mobility z	1×10^{-10}	1×10^{-5}	Log	$m^2V^{-2}s^{-1}$
Shunt resistance	parasitic/Shunt resistance	1.42×10^3	28.0	Log	Ω
Series resistance	parasitic/Series resistance	1.0	40.0	Lin	Ωm^{-2}
Electron Urbach energy	Electron tail slope	0.03	0.13	Lin	eV
Hole Urbach energy	Hole tail slope	0.03	0.13	Lin	eV
Electron trap density	Electron trap density	1×10^{18}	1×10^{26}	Log	m^{-3}
Hole trap density	Hole trap density	1×10^{18}	1×10^{26}	Log	m^{-3}
Electron capture cross-section	Free electron to Trapped electron	1×10^{-25}	1×10^{-18}	Log	m^2
Electron escape cross-section	Free electron to Trapped hole	1×10^{-25}	1×10^{-18}	Log	m^2
Hole capture cross-section	Free hole to Trapped electron	1×10^{-25}	1×10^{-18}	Log	m^2
Hole escape cross-section	Trapped hole to Free hole	1×10^{-25}	1×10^{-18}	Log	m^2

Table S1. Properties of the devices randomly varied in order to create the datasets used to train the Machine Learning Networks. "ActiveLayer" is used as a placeholder as particular to the material used in the active layer.

B. Point Prediction

1. Hyper-Parameters

Hyper-Parameter	Value
Initializer	he_normal
Activation	SiLU
Regularization	None
Layer Nodes	256
Number of Layers	4
Dropout	0.05
Epochs	4096
Patience	128
Batch Normalisation	True
Batch Size	1024
Loss Function	MSE
Metrics	MAE
Training Percentage	0.8
Initial earning Rate	1×10^{-4}
Decay Rate	0.8

Table S2. Hyper-Parameters used to train the point prediction networks

2. Confusion matrices

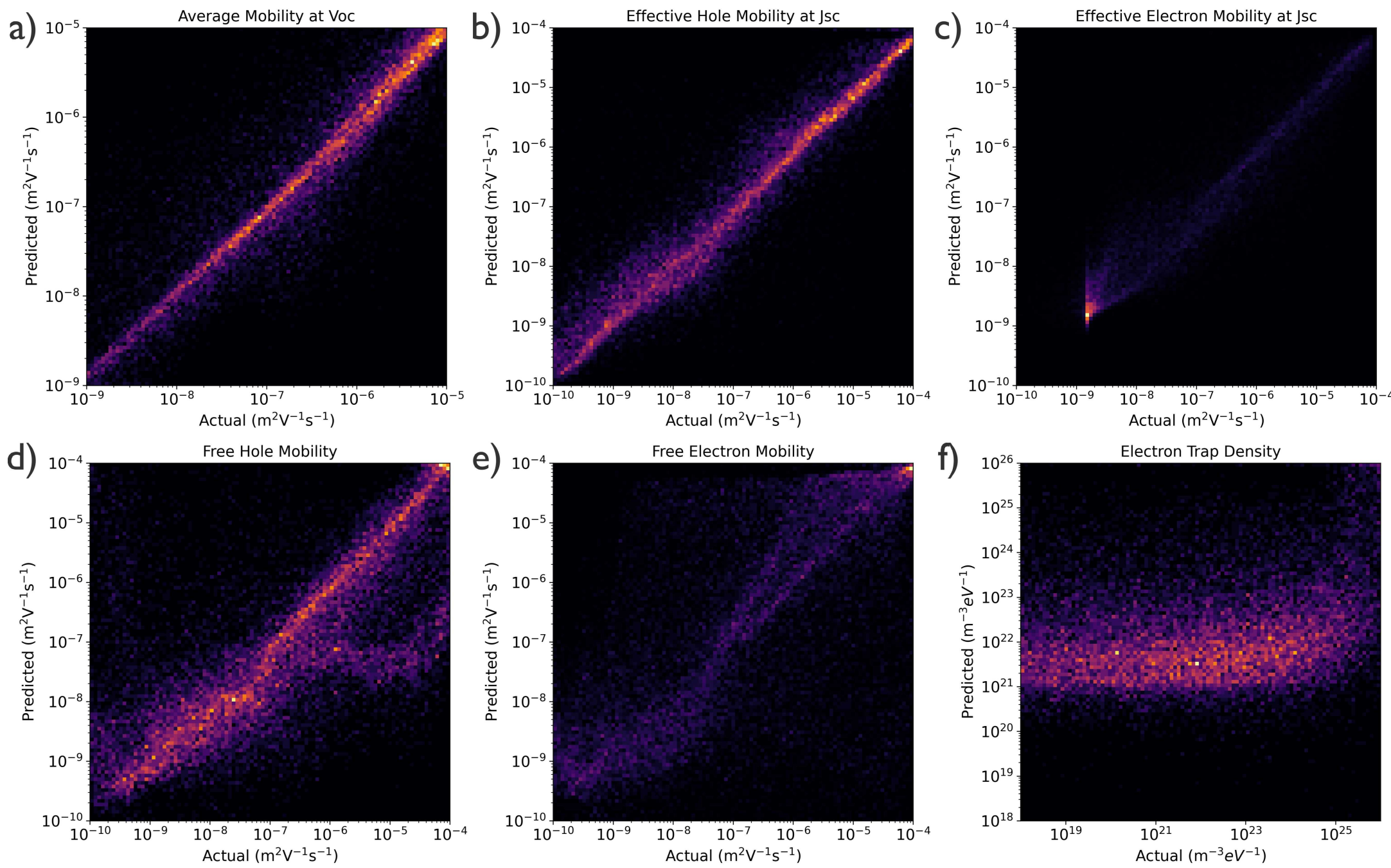

Figure S1. Confusion matrices from the Point method for: a) Average mobility at Voc; b) Effective Hole mobility at Jsc; c) Effective Electron Mobility at Jsc; d) Free Hole Mobility; e) Free Electron Mobility, and f) Electron Trap Density

C. Difference Method

1. Hyper-Parameters

Hyper-Parameter	Value
Initializer	he_normal
Activation	SiLU
Regularization	None
Layer Nodes	256
Number of Layers	4
Dropout	0.05
Epochs	1024
Patience	16
Batch Normalisation	True
Batch Size	16348
Permutations Limit	10×10^6
Loss Function	MSE
Metrics	MAE
Training Percentage	0.8
Initial earning Rate	1×10^{-5}
Decay Rate	0.8

Table S3. Hyper-Parameters used to train the difference method networks

2. Confusion Matrices

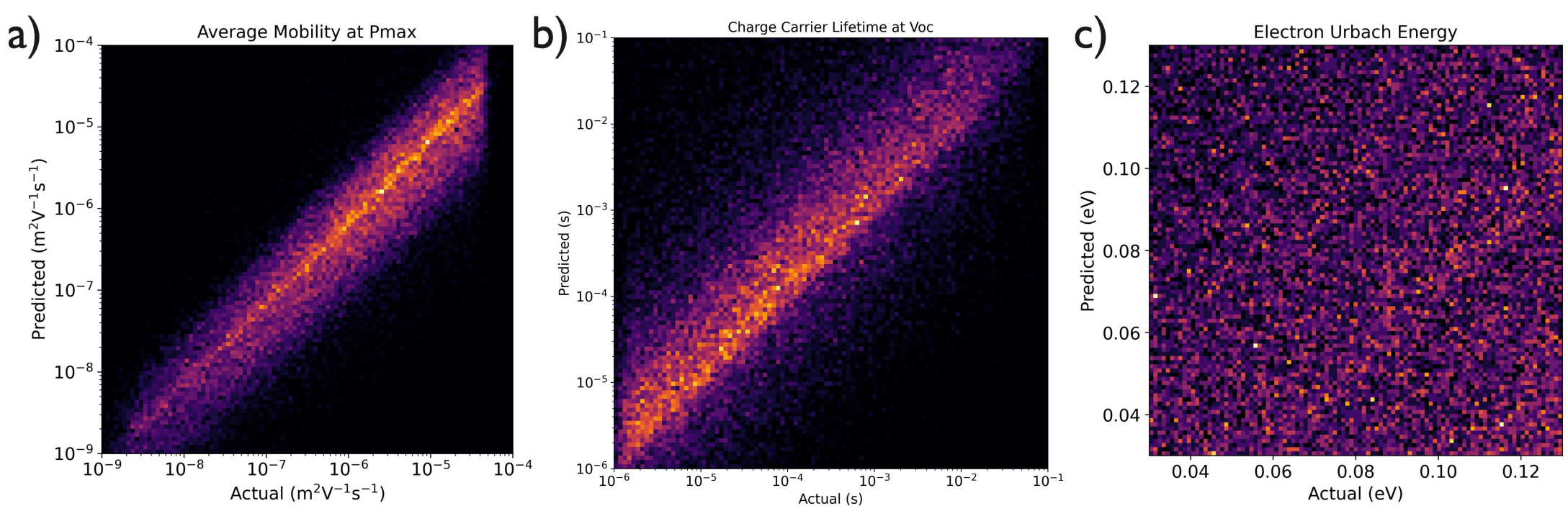

Figure S2. Confusion matrices for; a) Average mobility at the maximum power point μ_{pmax} ; b) Charge carrier lifetime at open circuit voltage τ_{voc}, and c) Electron Urbach Energy E_u^e.

D. Residual Method

1. Hyper-Parameters

**Hyper-Parameter**	**Value**
Initializer	he_normal
Activation	SiLU
Regularization	None
Layer Nodes	128
Number of Layers	4
Number of Residual Blocks	4
Dropout	0.05
Epochs	1024
Patience	16
Batch Normalisation	True
Batch Size	1024
Permutations Limit	10×10^6
Loss Function	MSE
Metrics	MAE
Training Percentage	0.8
Initial earning Rate	8×10^{-5}
Decay Rate	0.6

Table S4. Hyper-Parameters used to train the residual method networks

2. Confusion Matrices

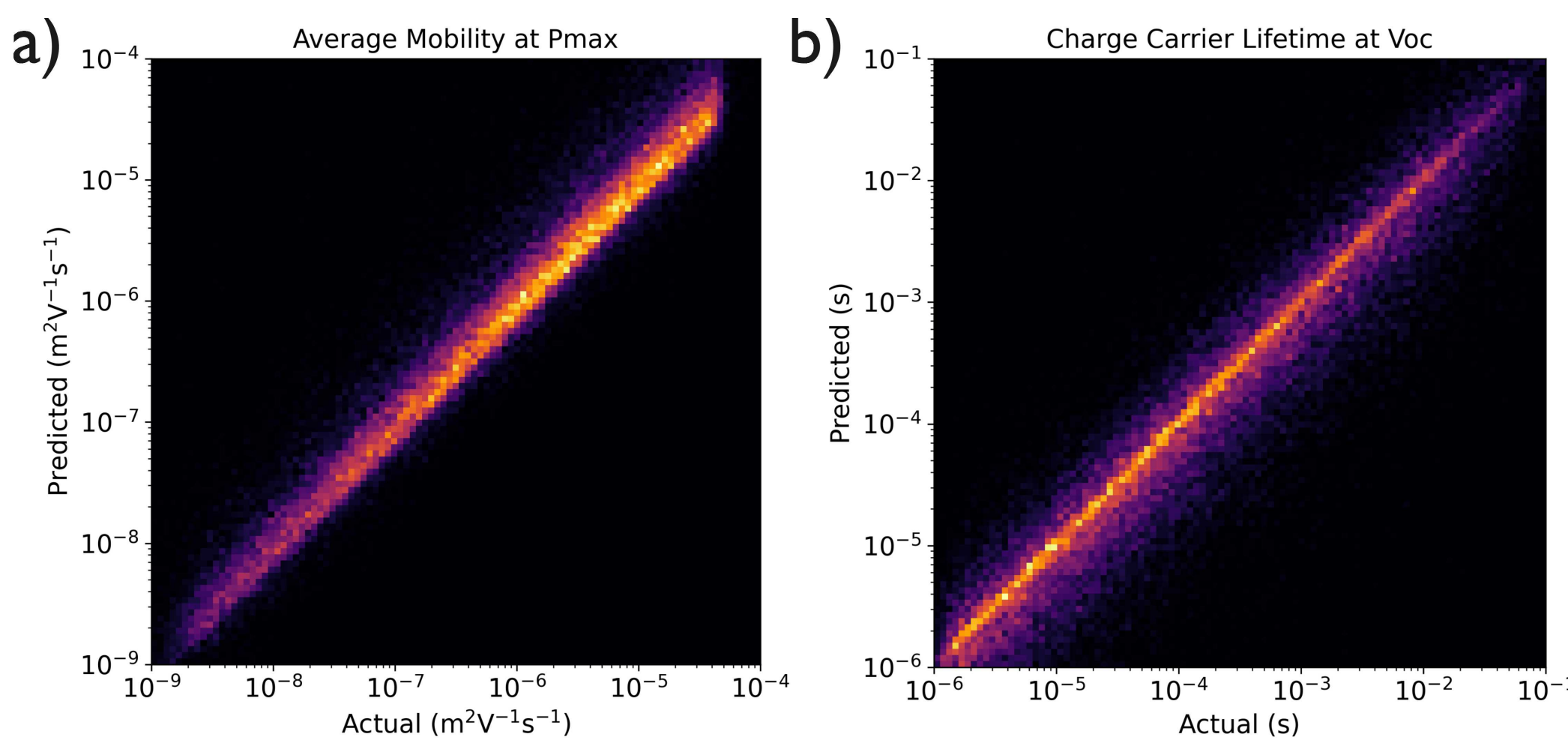

Figure S3. Confusion matrices for; a) Average mobility at the maximum power point μ_{pmax} , and b) Charge carrier lifetime at open circuit voltage τ_{voc}

E. Network Comparison

Property	Root Mean Squared Error	
	Difference	Residual
Effective Electron Mobility at Jsc under 1.0 Suns	1.806	1.14×10^{-3}
Effective Hole Mobility at Jsc under 1.0 Suns	0.032	1.02×10^{-5}
Electron Trap Density	2.49×10^{32}	1.76×10^{29}
Hole Trap Density	1.24×10^{32}	2.15×10^{31}
Electron Urbach Energy	0.060	0.029
Hole Urbach Energy	0.054	0.030

Table S5. Root Mean Squared Error (RMSE) values for the difference and residual networks presented within the paper. It can bee seen that overall the residual network achieves improved RMSE when compared to the respective difference network. A difference network has 226,049 trainable parameters, whilst a residual network has 1,007,233 trainable parameters.

S2. THE NEED TO INCLUDE TRAP STATES

It should be noted that when modelling disordered semiconductors, it is essential to use a model that explicitly accounts for carrier trapping and recombination through trap states. Disordered systems, such as organic semiconductors, exhibit a broad distribution of localized states. These trap states profoundly affect the charge carrier dynamics by capturing carriers, modulating their mobility, and enabling recombination processes that deviate from ideal band-to-band behaviour.

Neglecting trap states in a model can lead to a significant underestimation throughout the device. This is not a minor issue: the electrostatic potential profile within the device is governed by the spatial distribution of charge, and thus errors in carrier density will propagate directly into errors in the calculated internal electric field. This, in turn, affects carrier drift, diffusion, and ultimately the JV curve.

Moreover, key parameters such as the mobility, recombination rate, and charge carrier lifetime are all strongly dependent on the local carrier density. If the model fails to accurately reflect the carrier trapping, any inferred values for these parameters will be biased or unphysical. In particular, mobility can appear artificially low if trapping is neglected. Similarly, recombination rates may be forced to be incorrect to match experimental data.

Therefore, to make meaningful comparisons between simulation and experiment, and to extract physically accurate material parameters, the inclusion of trap-assisted processes is not optional - it is a requirement of any reliable model for disordered semiconductors.

S3. REPOSITORIES

The code utilised, along with documentation for its usage and installation, can be found in the GitHub repository: PyOghma_ML.

The datasets used for training the networks presented in this paper are available at the GitHub repository: OPV Machine Learning Datasets

S4. MATERIAL DATA

A. Device Fabrication

The patterned ITO substrates were pre-cleaned by ultrasonic baths for 15 minutes in diluted Hellmanex, deionized water, acetone, and 2-propanol sequentially, and then dried with a flowing nitrogen stream. ZnO nanoparticle dispersion (2.5 wt% in 2-propanol) was spin-coated onto the ITO substrate at 2500 rpm for 60 s, followed by thermal treatment at 200 °C for 30 minutes, resulting in a ZnO layer of approximately 37 nm.

The substrates were then transferred into a nitrogen-filled glove box for subsequent active layer and hole transport layer deposition. PM6:BTP-eC9 and PM6:Y12 solutions were prepared in advance, using identical formulations with a 1:1.2 donor-to-acceptor weight ratio. Chloroform was used as the solvent without any additives, with a total concentration of 12 mg/mL. The solutions were stirred at 600 rpm overnight and heated to 45 °C prior to deposition.

The warm solutions were spin-coated onto ITO/ZnO substrates at 3000 rpm for 30 s, followed by thermal annealing at 100 °C for 10 minutes. The resulting active layer thicknesses were approximately 50–60 nm.

An ethanol-based PEDOT:F solution was used as the hole transport layer. It was spin-coated dynamically at 3000 rpm for 50 s, followed by thermal treatment at 100 °C for 5 minutes.

Finally, the devices were completed by thermal evaporation of 150 nm silver (Ag) in a vacuum chamber (vacuum level $\sim 2 \times 10^{-6}$ Torr) using a shadow mask defining an active area of 0.04 cm^2.

B. Device Characterisation

The fabricated devices were characterised using a variety of techniques in order to reveal device properties, such as charge carrier mobility and recombination lifetime. Here are details as to their characterisation.

1. Current–voltage measurements

A Keithley 236 SMU was used for voltage application and current measurement. AM1.5G illumination was provided by a Wavelabs LS-2 solar simulator. No aperture was used. The illumination was kept switched on for two seconds per measurement to prevent the sample temperature from increasing. We measured from reverse bias to forward bias with no fixed sweep speed due to enabled autoranging. The measurements were conducted in a nitrogen-filled glovebox.

2. IMVS and IMPS

Modulated and continuous illumination was provided by an Omicron A350 diode laser with a center wavelength of 515 nm. A Zurich Instruments MFLI lock-in amplifier with MF-IA, MF-MD, and MF-5FM options was used to measure sample current and voltage as well as to provide voltage to modulate the laser. The illumination intensity was varied using neutral-density filters mounted in a Thorlabs motorized filter wheel FW102C combined with a continuously variable neutral-density filter wheel. For IMPS and IMVS measurements, the amplitude of modulated illumination was chosen to be 10% of the bias illumination intensity to ensure small perturbation conditions. Light intensity was continuously monitored using a Newport 818-BB-21 silicon photodetector. To ensure open-circuit conditions during IMVS measurements, a 10 MΩ input resistance was used, while for IMPS, the measurements were performed close to short circuit. There, the current was measured over the input resistance of 50 Ω.

The corresponding data are shown in Figures S4 and S5. The characteristic frequency $\omega_\mathbf{c}$ – for both IMVS and IMPS – was determined from the peak of the imaginary component and was used to evaluate the recombination lifetime τ_{rec}

(for IMVS) and charge carrier mobility μ (for IMPS). The recombination lifetime was evaluated according to:[45]

$$\frac{\mathrm{d}\ln\omega_{\mathbf{c}}(\Phi)}{\mathrm{d}\ln\Phi} = 1 - \frac{1}{\delta}, \qquad \tau_{\mathrm{rec}} = \frac{\delta}{\omega_{\mathbf{c}}}. \tag{S1}$$

Here, the recombination order δ was determined using the light intensity-dependence of the characteristic frequency $\omega_{\mathbf{c}}(\Phi)$.

For IMPS, the mobility μ was evaluated according to:[46]

$$\mu = \frac{\omega_c L^2}{2 V_{\mathrm{bi}}}. \tag{S2}$$

Here, L is the thickness of the photoactive layer, and V_{bi} is the build-in voltage, estimated as 1.15 V.

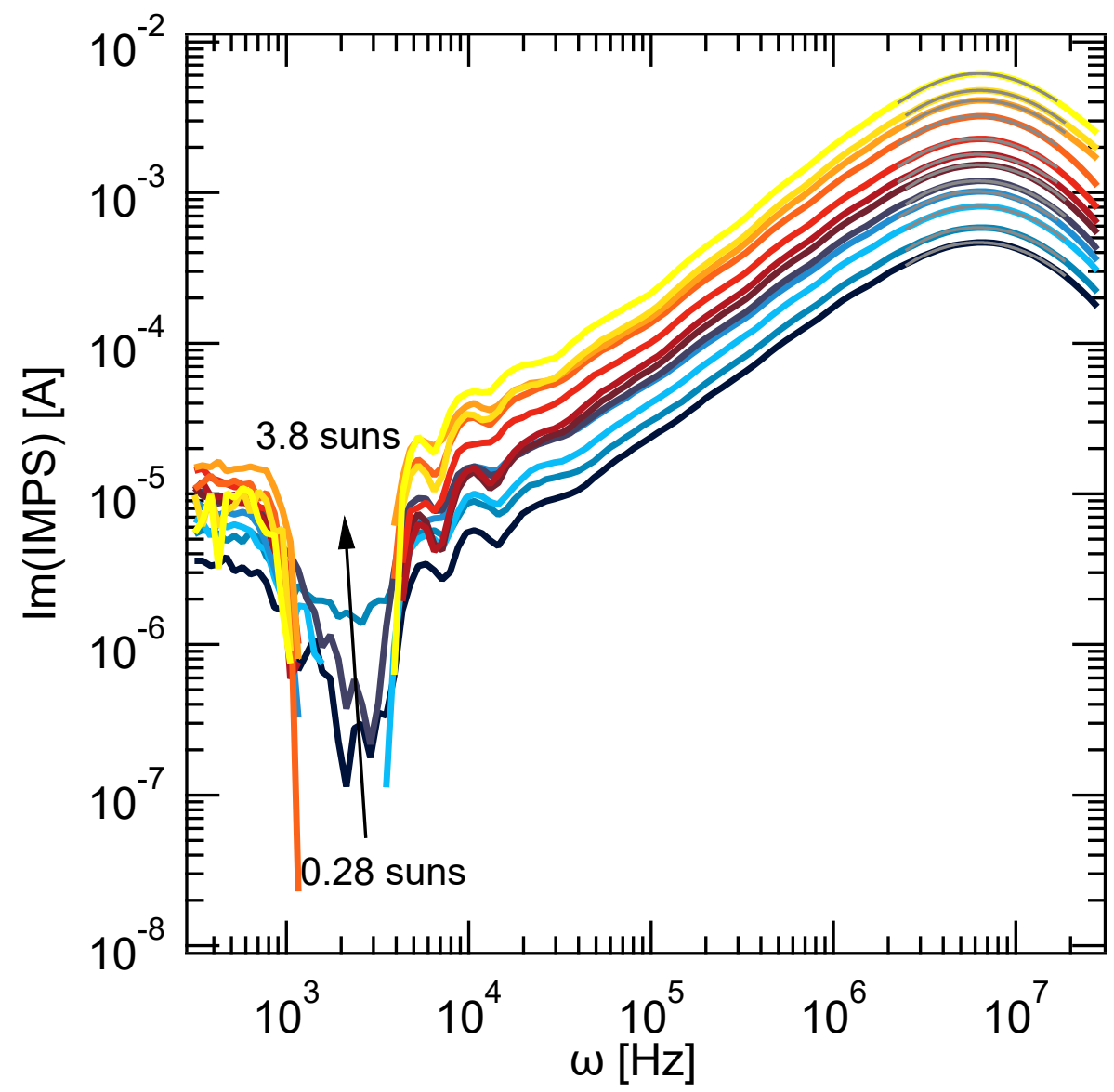

Figure S4. Imaginary part of experimental IMPS data with Gaussian fits indicated by grey lines at each respective peak.

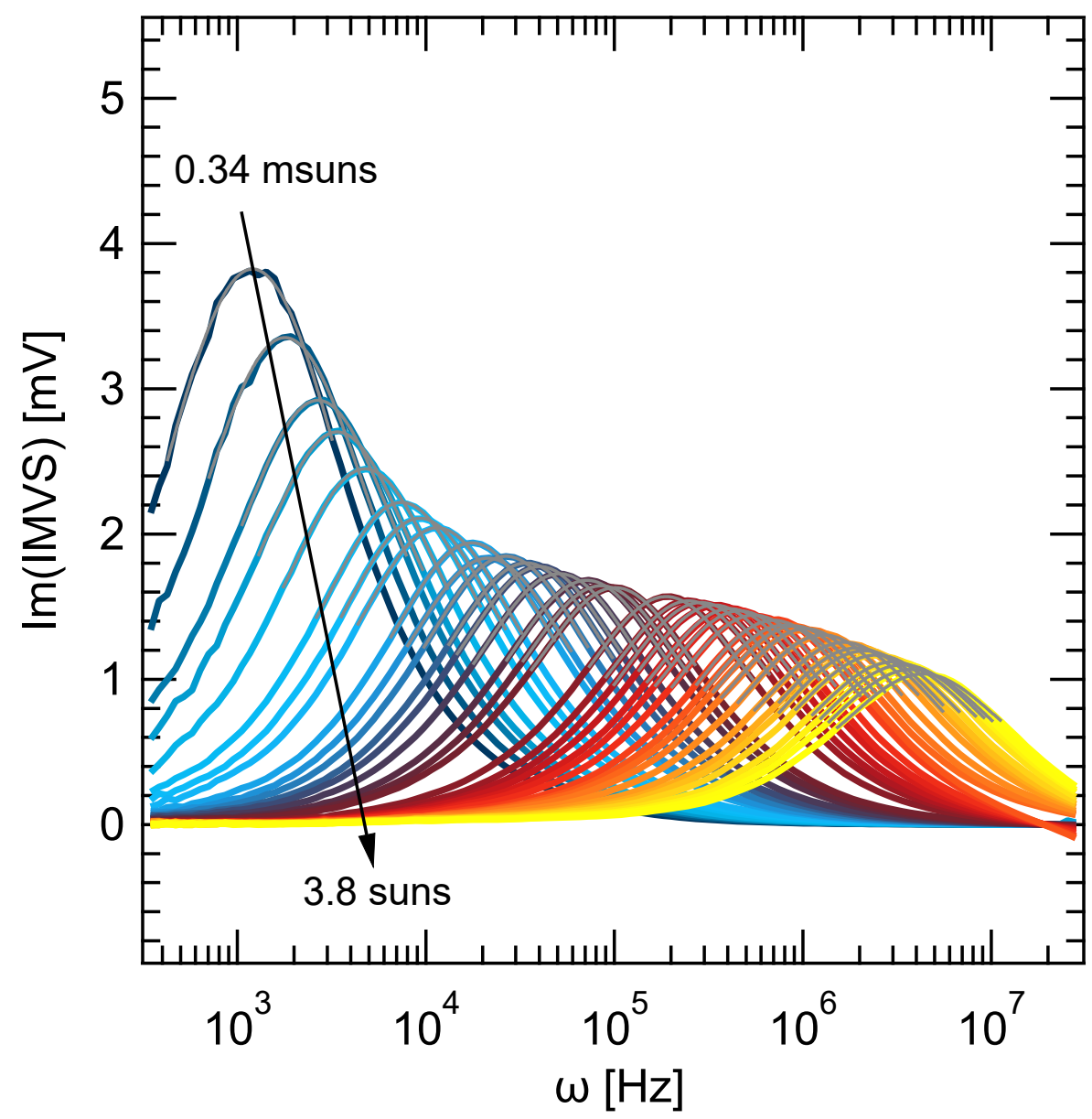

Figure S5. Imaginary part of experimental IMVS data with Gaussian fits indicated by grey lines at each respective peak.

3. SCLC

The hole- and electron-only devices were fabricated to determine the charge carrier mobility. The configuration of ITO/PEDOT:PSS/Active layer/PEDOT:F/Ag and ITO/ZnO/Active layer/PDINO/Al was used for the hole-only and electron-only devices, respectively. The current was measured using a Keithley 236 SMU, which was also used to apply voltage to the samples. The corresponding data are shown in Figures S6 and S7. The mobilities were determined using the Mott-Gurney equation:[47]

$$J = \frac{9}{8}\epsilon_r \epsilon_0 \mu_{e,h} \frac{V^2}{L^3}, \tag{S3}$$

where J is the current density, ϵ_r is the relative permittivity, assumed to be 3.5, ϵ_0 is the vacuum permittivity, $\mu_{e,h}$ is the mobility of electrons (e) or holes (h), V is the applied voltage, and L is the active layer thickness.

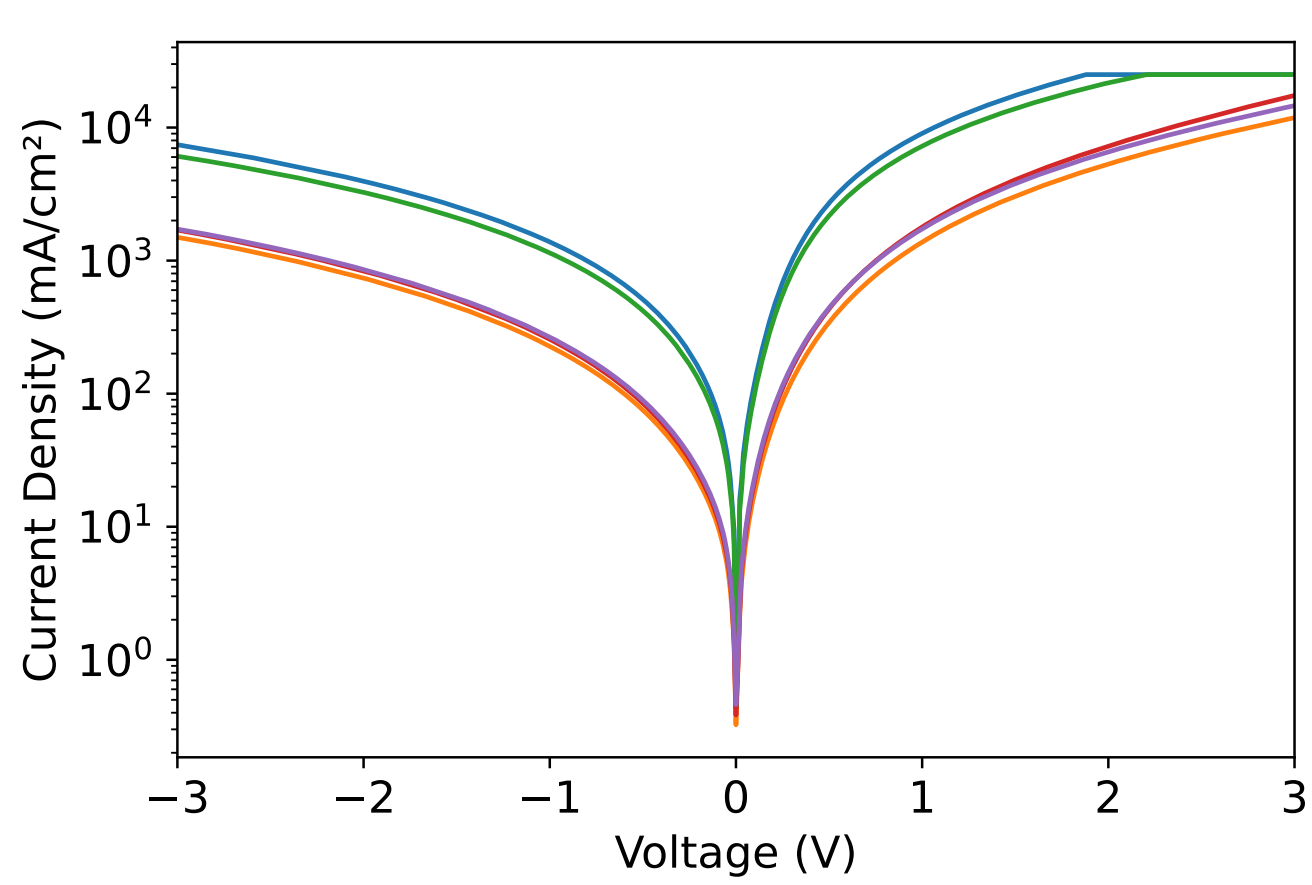

Figure S6. PM6:Y12 blend-based electron only device, different colours represent different pixels of the device.

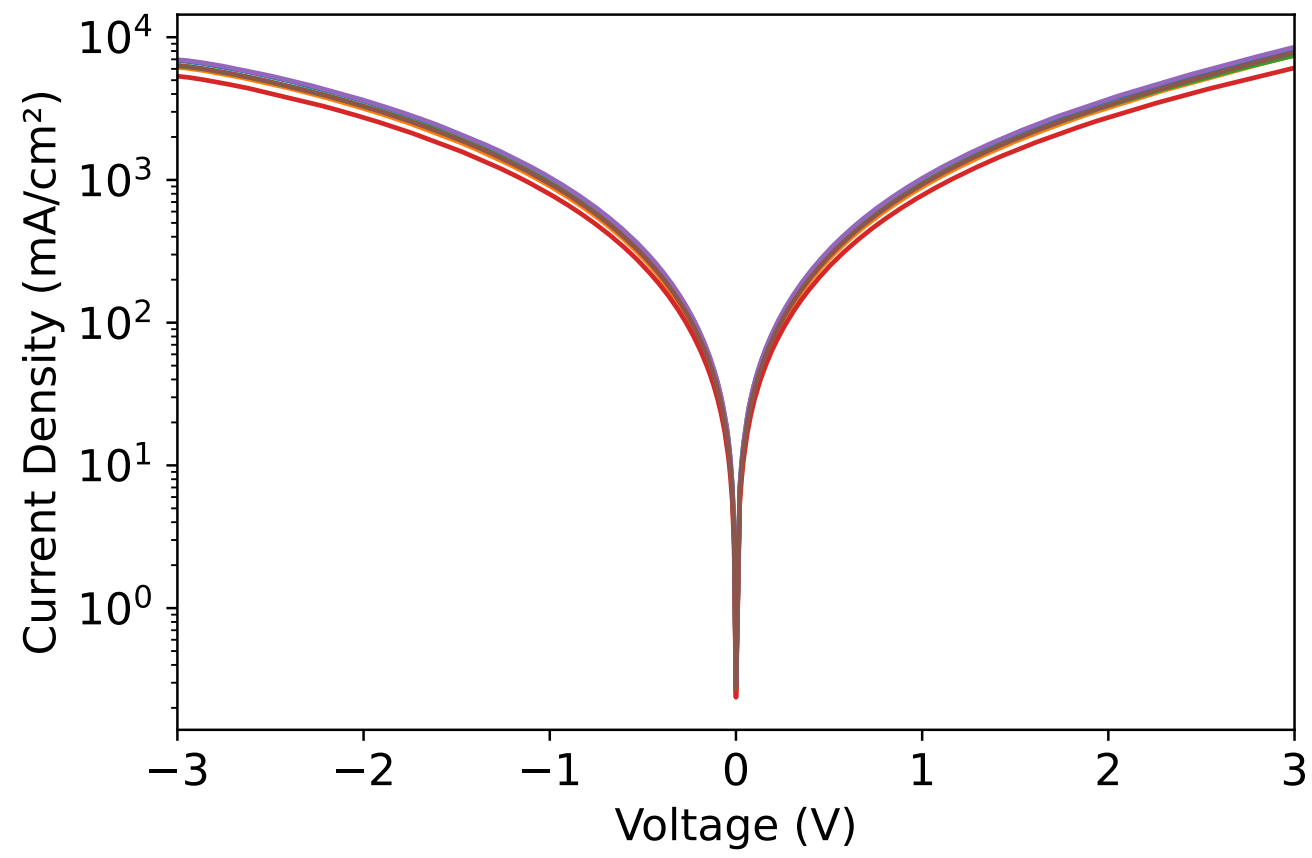

Figure S7. PM6:Y12 blend-based hole only device, different colours represent different pixels of the device.

4. Refractive Index and Extinction Coefficient

The optical constants n and k of ZnO, PEDOT:F, as well as PM6:Y12 and PM6:BTP-eC9 blend films were determined using variable-angle spectroscopic ellipsometry (VASE) measurements of individual layers deposited on glass substrates. The backsides of these samples were roughened before measurements to suppress the effect of backside reflections. The VASE measurements were performed with an M2000 T-Solar ellipsometer by J. A. Woollam at 7 different angles between 45° - 75° in steps of 5° and in a spectral range from approximately 0.7 - 5.1 eV, as provided by a Xe-arc lamp. The measurement area is between 200 – 300 µm depending on the angle of incidence. Three arbitrary but well-separated spots were measured on each sample to be correlated in the processing of the measurement data. The values of the optical constants of the thin films were extracted from a fitted optical model of the dielectric functions using the CompleteEASE Software by J. A. Woollam. To that end, a reference glass substrate was measured as well.

The modelling approach for all materials involved an initial approximation of the spectral range below the main absorption onset using a Cauchy function, as estimated from the line shape of the measured data. For the thin films, this also yielded an initial guess of the film thickness and surface roughness. Thereafter, absorption was first approximated via the reparametrisation of the imaginary part of the dielectric function using a B-Spline function from which the real part of the dielectric function was calculated via a Kramers–Kronig transformation. The resulting line shapes of the dielectric function were then parametrised using oscillator models. For the main absorption onsets, a Cody–Lorentz oscillator function was used. All other absorption features were fitted with Gaussian oscillators.

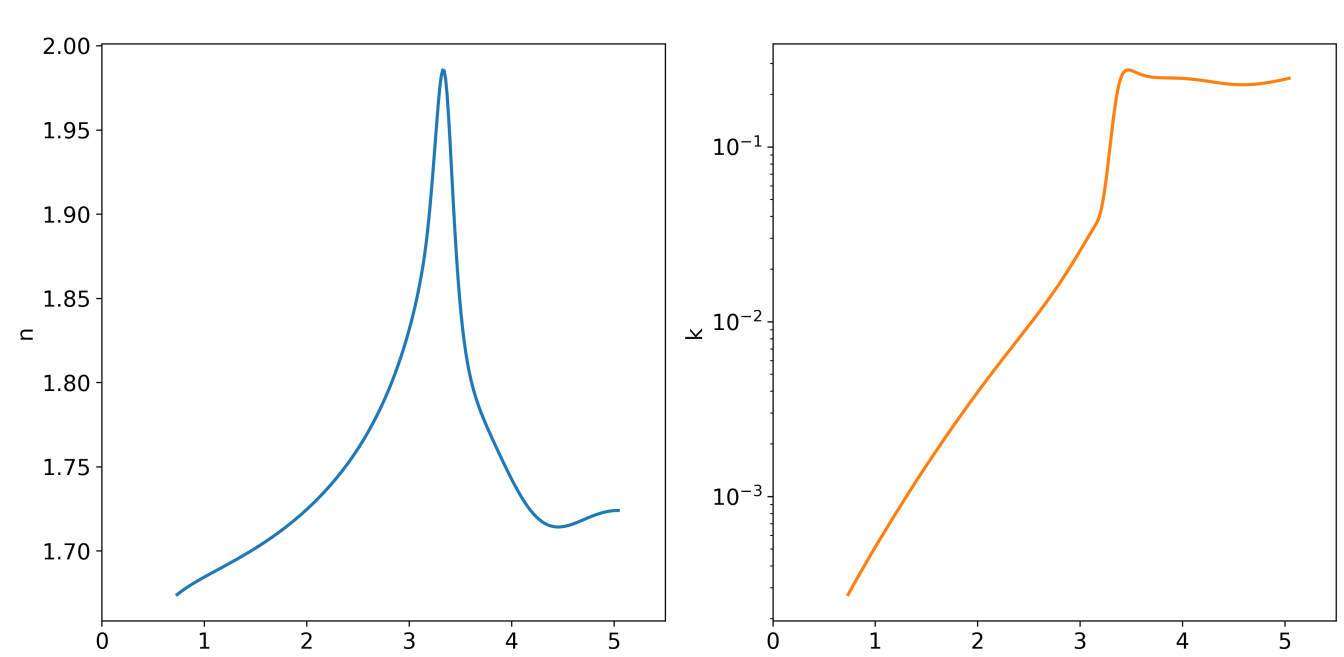

Figure S8. Refractive index (n) and extinction coefficient (k) data for a ZnO thin film.

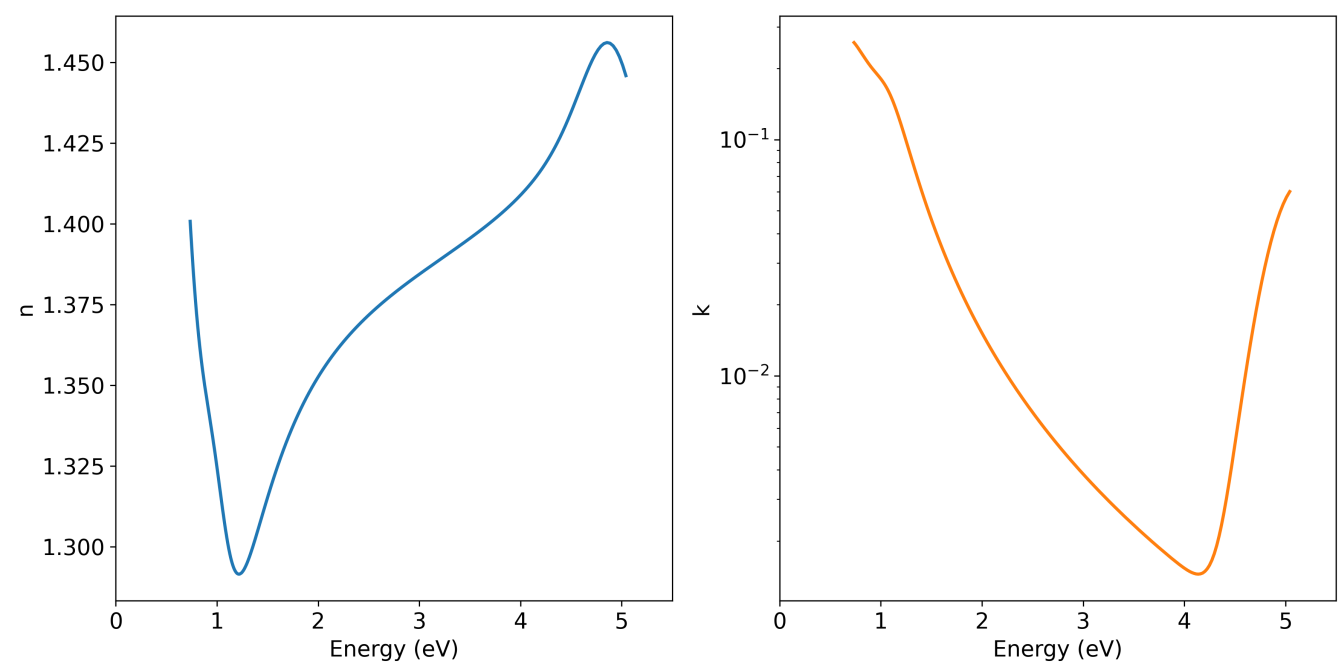

Figure S9. Refractive index (n) and extinction coefficient (k) data for a PEDOT:F thin film.

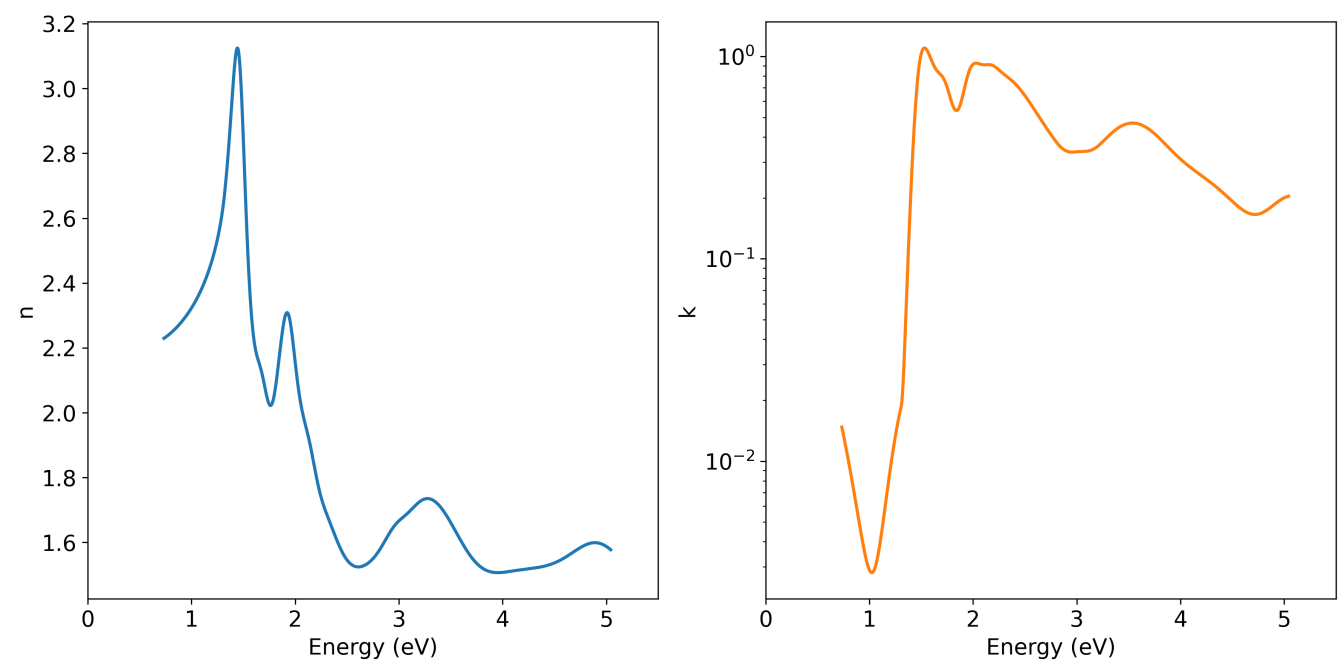

Figure S10. Refractive index (n) and extinction coefficient (k) data for a PM6:BTP-eC9 thin film.

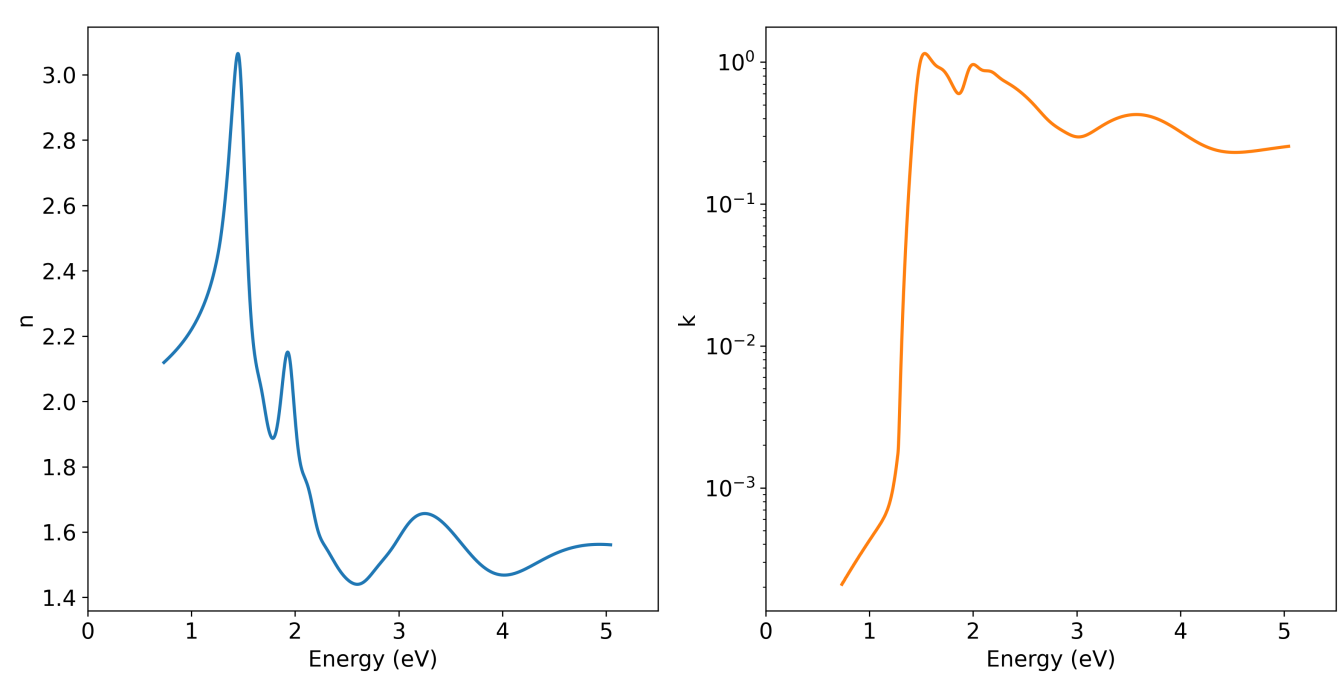

Figure S11. Refractive index (n) and extinction coefficient (k) data for a PM6:Y12 thin film.
